# Molecular docking analysis of vascular endothelial growth factor receptor with bioactive molecules from Piper longum as potential anti-cancer agents

**DOI:** 10.6026/97320630017223

**Published:** 2021-01-31

**Authors:** Selvaraj Jayaraman, Vidhya Rekha Umapathy, Jayamathi Govindaraj, Keerthidaa Govidaraj

**Affiliations:** 1 Department of Biochemistry, Saveetha Dental College and Hospitals, Saveetha Institute of Medical and Technical Sciences, Saveetha University, Chennai - 600 077, India; 2 Department of Public Health Dentistry, Sree Balaji Dental College and Hospital, Pallikaranai,Chennai-600 100, India; 3 Department of Biochemistry, Sree Balaji Dental College and Hospital, Pallikaranai, Chennai-600 100, India; 4 MGM Health Care, Nelson Manickam Road, Chennai- 600029, India

**Keywords:** VEGFR, Piper longum, lung cancer, molecular docking

## Abstract

It is known that vascular endothelial growth factor receptor (VGFR) is linked with cancer. Therefore, it is of interest to document the molecular binding features of bioactive molecules from Piper longum as potential anti-cancer agents with VGFR2 for further consideration. Thus, we document the binding features of four compounds (sesamin, fargesin, longamide and piperlonguminine) with VGFR2 for further consideration in drug discovery.

## Background:

The most common malignant tumour is lung cancer. An estimated 1.82 million people have been diagnosed with lung cancer and 1.6 million deaths worldwide have been linked to lung cancer in 2012 [1]. For advanced lung cancer, the tumour response rate (tRR) of standard platinum-based chemotherapy is only 25-35 percent, with a median overall survival (mOS) of 8-10 months [2]. Angiogenesis is significant in the development of tumors [3]. The most important pro-angiogenic factor is the Vascular Endothelial Growth Factor (VEGF). A host of stimuli, including oestrogen, nitric oxide and a variety of growth factors, are up-regulated by the VEGF gene, such as platelet growth factor, tumour necrosis factor alpha (TNF-alpha), fibroblast growth factor-4, keratinocyte growth factor, epidermal growth factor (EGF), interleukin (IL-6 and IL-1) [4]. The expression of VEGF is sensitive to the presence of oxygen and is mediated by hypoxia, which is due to the aberrant nature of the vascular supply of most tumours. In various cancers, such as colorectal cancer, breast cancer, non-small cell lung cancer, renal cell cancer, pancreatic cancer, prostate cancer, cancer of the head and neck, gynaecological cancer and haematological malignancies, VEGF plays an important role [5]. For various reasons, the VEGF pathway is a good target for anti-angiogenic therapy, such as: it is generated by growing primary tumours in large quantities; the VEGF pathway induces the production of sprouting blood vessels [6]; the VEGF pathway binds to endothelial cells involved in the formation of blood vessels; the endothelial cells are also genetically stable and spontaneous mutations are rare compared to unstressed mutations. Due to genomic stability, endothelial cells are considered an ideal target for therapies directed against cancer cells [8, 9]. VEGF is secreted from the over-expressive tumour and binds to signalling receptors of high affinity on the endothelial cells of existing blood vessels, leading to the development of new blood capillaries that provide the necessary nutrients for the survival and growth of tumour cells [10]. Therefore, it is of interest to document the molecular binding features of bioactive molecules from Piper longum as potential anti-cancer agents with VGFR2 for further consideration.

## Materials and Methods

### Protein Preparation:

The three-dimensional (3D) crystal structures of Vascular endothelial growth factor receptor VEGFR (PDB ID: 1FLT) was downloaded from the Protein Data Bank (PDB) (www.pdb.org/pdb). The protein structure was then refined for docking using the Chimera© (version 1.13) software tool (http://www.cgl.ucsf.edu/chimera).

### Ligand Preparation:

22 compounds in Piper longum were downloaded from the PubChem database (https://pubchem.ncbi.nlm.nih.gov/) in SDF format using information gleaned from literature and converted to the PDB format using the Online Smiles Translator.

### Molecular docking:

The molecular screening was done using the PyRx autodock wizard software [11,12]. The docking was completed using the flexible docking protocol as described by Trott & Olson, (2005) with modifications as described by Sekar et al. The results were further analyzed using PYMOL.

## Results and Discussion:

**Figure 1:**
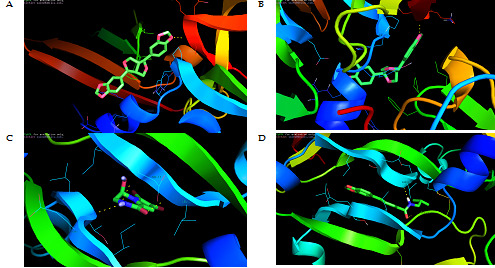
Molecular docking interaction of VEGF with (a) Sesamin, (b) Fargesin, (c) Longamide and (d) Piperlonguminine.

Vascular endothelial growth factor receptor has been found in high concentrations in various cancer diseases and is known as targets for anticancer agents, as mentioned earlier. Docking is the process within the active site of a protein receptor with a known structure that identifies the best binding conformation or poses for a ligand [13,14]. Molecular docking was therefore carried out on VEGFR with the phytocompounds in the present study to elucidate their interactions and to obtain additional information from all the compounds in molecular binding mode. The molecular docking results include appropriate interactions of the chemical constituents with the main residues of amino acids at the protein active site. The results of molecular docking between the active compounds assessed with VEGFR are shown in Table 2 (see PDF) and Figure 1. The results of the docking indicate that all piperlongum bioactive compounds show better binding positions with VEGFR. For docking studies, 22 piperlongum compounds were selected in total. The strong binding in terms of binding power and hydrogen bonding was shown by compound Sesamin among them. Sesamin's binding energy is -7.4 kcal.mol. We have selected three other compounds based on the binding energy, namely Fargesin (-6.9kcal/mol), Longamide (-6.6kcal/mol), Piperlonguminine (-6.1). All four of these compounds demonstrated strong binding with VEGFR (Table 2 - see PDF). Molecular docking analysis has shown that the selected bioactive compounds have interacted with a different binding mode at a similar site. These bioactive compounds have established hydrogen interactions at the VEGFR binding site with CYS-102, HIS-27, CYS-102, GLU-30, THR-31, LEU-32, LEU-32 and GLY-59. The interaction of this bioactive compound with the amino acids listed above indicates that the compound has penetrated deeply into the VEGFR binding site. (Figure 1) This could be the reason why it also had the lowest binding energy to inhibit the role of VEGFR in angiogenesis. It is clear from the results that these phytocompounds had relatively good binding energy and, just like the standard drug, they were able to bind to VEGF.

## Conclusion

We document the binding features of four compounds (sesamin, fargesin, longamide and piperlonguminine) with VGFR2 for further consideration in drug discovery.
